# Editorial: Advances in morphogenesis and patterning: zebrafish as a model organism

**DOI:** 10.3389/fcell.2024.1376663

**Published:** 2024-02-02

**Authors:** Maria Iribarne

**Affiliations:** ^1^ Department of Biological Sciences, University of Notre Dame, Notre Dame, IN, United States; ^2^ Center for Stem Cells and Regenerative Medicine, University of Notre Dame, Notre Dame, IN, United States; ^3^ Center for Zebrafish Research, University of Notre Dame, Notre Dame, IN, United States

**Keywords:** developmental biology, morphogenesis, zebrafish, zebrafish disease model, gene mutations, transgenic lines

The precise regulation of morphogenesis and patterning is crucial for generating the appropriate type and proportion of different cell types at the right time to ultimately produce a healthy organism. Zebrafish has proved to be a valuable animal model to understand these processes during development and disease. Zebrafish possesses several practical features that ensure its popularity as an animal model, including its small size, ease of maintenance, large offspring number, and short generation time. The transparency of zebrafish embryos, coupled with an extensive Research Topic of transgenic lines, facilitates non-invasive live imaging. Importantly, zebrafish shares 75% of gene homology with humans, and approximately 85% of disease-causing genes having at least one zebrafish orthologue. Due to their external development, genetic modifications like knockdown or knockout experiments are easy to perform.

In this Research Topic, we present various research and review articles that illustrate how zebrafish can serve as an animal model to unveil the molecular and cellular mechanism underlying the morphogenesis and patterning in humans. For instance, disruption in the FOXE1 gene induces cleft palate and thyroid malformation in humans. In their research paper, Raterman et al. investigated the role of foxe1 during the development of the craniofacial skeleton and the thyroid in zebrafish. Foxe1 is widely expressed, including in oral epithelium, ethmoid plate, eyes, and thyroid follicles. The *foxe1* mutant embryos generated for this study showed abnormal mineralization of the bones and neural crest cell dysfunctions, while the thyroid morphology remained unaffected. Foxe1 regulates chondrogenesis and osteogenesis genes during early development such as *sp7*, *col2a1*, and *sox9a*. This research demonstrated the conserved role of Foxe1 in the skeletal development in zebrafish and humans at early developmental stages.

Another gene associated with cleft palate in humans is FOS. Here Maili et al. explored the role of Fos in zebrafish craniofacial development through genetic disruption and knockdown approaches. Fos was detected in craniofacial tissues during zebrafish development, and fos morphants and mutants have abnormal craniofacies, with a distinctive abnormal ovoid oral cavity shape. Genetic perturbation of *fos* also causes epithelial abnormalities, an increase in cell apoptosis followed by a reduction in cranial neural crest cell numbers that led to abnormalities in bone, cartilage, and tooth development. Additionally, a reduction in Wnt-responsive cells around the oral cavity was observed. Fos might play a critical regulatory role in guiding the interaction between the oral epithelial and mesenchymal cells through the Wnt/β-catenin pathway.

The study by Belmonte-Mateos et al. and collaborators focused on the head of the development of zebrafish but in the case of the hindbrain rhombomeres, which are formed by segmentation of the embryonic hindbrain. Using cell lineage and *in vivo* imaging techniques the authors characterized the cells in the center of the rhombomeres. They found that these cells are progenitor cells with a molecular expression and proliferative capacity that changes during development. Furthermore, the Notch3 signaling regulates these cells as non-committed progenitors.

Lastly, Vöcking and Famulski studied in their review article how single cell transcriptome analyses are helping to move forward our current molecular knowledge in the developing zebrafish eye. This mini-review covers many of the advantages of using zebrafish for single cell transcriptome of developmental processes, such as samples accessibility, the ability to recover a high number of embryos and determined tissues at specific developmental point times, when combined with transgenic lines it is possible to isolate and analyze specific cell types, and candidates genes identified with single cell transcriptome analysis can be easily characterized using highly efficient genetic knockouts methods.

In conclusion, this Research Topic on *Advances in Morphogenesis and Patterning: Zebrafish as a Model Organism* contains a Research Topic of high-quality research and review papers that enlighten the usefulness of zebrafish as a model organism. Overall, a more in-depth studies of molecular pathways, the integration of cutting-edge technologies, and a comprehensive understanding of environmental influences are likely to shape the future trajectory of morphogenesis and patterning studies in zebrafish. We trust that the data and information we have shared will prove valuable to the scientific community.

